# Biological and Biochemical Roles of Two Distinct Cyclic Dimeric Adenosine 3′,5′-Monophosphate- Associated Phosphodiesterases in *Streptococcus mutans*

**DOI:** 10.3389/fmicb.2018.02347

**Published:** 2018-09-27

**Authors:** Hiroyasu Konno, Yasuo Yoshida, Keiji Nagano, Jun Takebe, Yoshiaki Hasegawa

**Affiliations:** ^1^Department of Microbiology, Aichi Gakuin University School of Dentistry, Nagoya, Japan; ^2^Department of Removable Prosthodontics, Aichi Gakuin University School of Dentistry, Nagoya, Japan

**Keywords:** cyclic di-AMP, phosphodiesterase, *Streptococcus mutans*, biofilm, pApA

## Abstract

Cyclic dimeric adenosine 3′,5′-monophosphate (c-di-AMP), a recently identified secondary messenger in bacteria, plays a role in several bacterial processes, including biofilm formation. It is enzymatically produced by diadenylate cyclase and cleaved by c-di-AMP phosphodiesterase. c-di-AMP is believed to be essential for the viability of bacterial cells that produce it. In the current study, the biochemical and biological roles of GdpP (SMU_2140c) and DhhP (SMU_1297), two distinct *Streptococcus mutans* phosphodiesterases involved in the pathway producing AMP from c-di-AMP, were investigated. Liquid chromatography-tandem mass spectrometry revealed that c-di-AMP was degraded to phosphoadenylyl adenosine (pApA) by truncated recombinant GdpP, and pApA was cleaved by recombinant DhhP to yield AMP. In-frame deletion mutants lacking the *dhhP* gene (Δ*dhhP*) and both the *gdpP* and *dhhP* genes (Δ*gdpP*Δ*dhhP*) displayed significantly more biofilm formation than the wild-type and a mutant strain lacking the *gdpP* gene (Δ*gdpP*; *p* < 0.01). Furthermore, biofilm formation was restored to the level of the wild type strain upon complementation with *dhhP*. Optical and electron microscopy observations revealed that Δ*dhhP* and Δ*gdpP*Δ*dhhP* mutants self-aggregated into large cell clumps, correlated with increased biofilm formation, but cell clumps were not observed in cultures of wild-type, Δ*gdpP*, or strains complemented with *gdpP* and *dhhP*. Thus, deletion of *dhhP* presumably leads to the formation of bacterial cell aggregates and a subsequent increase in biofilm production.

## Introduction

*Streptococcus mutans* is a major etiological agent of dental caries in humans (Hamada and Slade, 1980), a biofilm-associated infectious disease and important health problem worldwide (Thenisch et al., 2006). This microorganism has the capacity to prevail in the complex microbial community of an oral biofilm under the low pH conditions responsible for tooth demineralization, and can adapt to the stressful conditions to which the cariogenic biofilms are exposed (Lemos and Burne, 2008).

Bacterial second messengers, including cyclic dimeric nucleotides, are molecules that control important signaling cascades in bacteria and their hosts during infection (Pesavento and Hengge, 2009). Cyclic dimeric guanosine 3′,5′-monophosphate (c-di-GMP), a signaling molecule that acts as a secondary messenger, is important for control of biofilm formation, adhesion, motility, virulence, and cell morphogenesis (Boyd and O’Toole, 2012). This molecule is synthesized from two molecules of guanosine 3′,5′-triphosphate (GTP) by a Gly-Gly-Asp-Glu-Phe (GGDEF) domain-containing diguanylate cyclase, and hydrolyzed to phosphoguanylyl guanosine by phosphodiesterase enzymes containing a highly conserved Glu-Ala-Leu (EAL) domain (Mills et al., 2011) or a His-Asp-Gly-Tyr-Pro (HD-GYP) domain (Hengge, 2009).

Cyclic dimeric adenosine 3′,5′-monophosphate (c-di-AMP) has recently emerged as a widely conserved secondary messenger (Witte et al., 2008). This molecule is thought to be essential for the viability of bacterial cells that produce it. Interestingly, Gram-positive bacteria mostly synthesize c-di-AMP (Woodward et al., 2010; Corrigan et al., 2011; Kamegaya et al., 2011; Bai et al., 2013; Du et al., 2014). c-di-AMP has been found to regulate many important function in bacteria, including the homeostasis of cell wall peptidoglycan architecture (Luo and Helmann, 2012), size and envelope stress (Corrigan et al., 2011), biofilm formation (Corrigan et al., 2011; Diethmaier et al., 2011; Du et al., 2014; Peng et al., 2016), potassium uptake (Bai et al., 2014), metabolic enzyme function (Sureka et al., 2014), and drug resistance (Banerjee et al., 2010; Corrigan et al., 2011; Griffiths and O’Neill, 2012; Luo and Helmann, 2012; Cho and Kang, 2013). This molecule also reportedly couples DNA integrity with the sporulation progression, cell growth, competence development, and spore resurrection (Oppenheimer-Shaanan et al., 2011). However, these functions of c-di-AMP are not necessarily shared by all Gram-positive bacteria. This molecule also appears to trigger the cytosolic pathway of innate immunity (Woodward et al., 2010; Parvatiyar et al., 2012; Dey et al., 2015).

c-di-AMP is enzymatically produced by diadenylate cyclases and cleaved by c-di-AMP phosphodiesterases. Diadenylate cyclases typically contain a diadenyl cyclase domain that is crucial for the synthesis of c-di-AMP from two molecules of ATP (Corrigan and Grundling, 2013), while phosphodiesterases degrading c-di-AMP are divided into two distinct classes. The first class is characterized by a catalytically active Asp-His-His/Asp-His-His-Ala (DHH/DHHA1) domain, whereas the second class contains a His-Asp (HD) domain in the active center (Huynh et al., 2015). DHH/DHHA1 domain-containing proteins responsible for c-di-AMP degradation are further divided into two subfamilies. The first subfamily comprises homologs of membrane-bound GdpP proteins that contain two N-terminal transmembrane helices, a Per-Arnt-Sim (PAS) domain, a degenerate GGDEF domain, and a catalytic DHH/DHHA1 domain (Rao et al., 2010). PAS domains usually function as sensory domains for detecting light, redox potential, and oxygen (Taylor and Zhulin, 1999). However, the domain appears to function for GdpP, since GdpP without the PAS domain was enzymatically equivalent to that with the domain (Bai et al., 2013). Proteins belonging to the other subfamily are homologs of the soluble DhhP protein from *Borrelia burgdorferi* that contains only a cytosolic core DHH/DHHA1 domain (Ye et al., 2014). It is interesting that *Streptococcus pneumoniae* harbors two DHH/DHHA1 domain-containing phosphodiesterases from both subfamilies, named Pde1 and Pde2 (Bai et al., 2013), which are orthologues of GdpP and Dhhp. The enzymatic activity of Pde1 is the same as that of membrane-bound GdpP proteins degrading c-di-AMP to pApA, whereas Pde2 cleaves both c-di-AMP and pApA to yield AMP. A recent study revealed that deletion of the *gdpP* gene (SMU_2140c) in *S. mutans* increased both the intracellular c-di-AMP concentration and biofilm production in the presence of sucrose (Peng et al., 2016). Since no ortholog of Pde2 in *S. mutans*, Dhhp (SMU_1297), has yet been fully investigated, the second putative phosphodiesterase should be characterized. In the current study, the *S. mutans* genes *gdpP* and *dhhP* were expressed in *Escherichia coli*, and the recombinant proteins were purified and enzymatically characterized. Markerless in-frame deletion mutant strains, including a double mutant strain, were constructed, and phenotypic characterization of these mutant strains was performed to verify the functions of these proteins.

## Materials and Methods

### Bacterial Strains and Growth Conditions

*Streptococcus mutans* XC (Koga et al., 1989) and its derivatives (**Table 1**) were anaerobically grown in Brain Heart Infusion broth (BHI; Becton Dickinson, Franklin Lakes, NJ, United States) at 37°C. Where necessary, erythromycin, kanamycin, or 4-chloro-phenylalanine (*p*-Cl-Phe; Sigma-Aldrich Japan, Tokyo, Japan) was added to a final concentration of 20 μg ml^-1^, 700 μg ml^-1^, or 200 μg ml^-1^, respectively. *E. coli* strains DH5α (Thermo Scientific Japan, Tokyo, Japan) and BL21 (Promega, Tokyo, Japan) were used for DNA manipulation and recombinant protein purification, respectively, and were grown aerobically in 2 × YT broth (Becton Dickinson) at 37°C. Where required, chloramphenicol, erythromycin, ampicillin, and kanamycin were added to the media to final concentrations of 20 μg ml^-1^, 200 μg ml^-1^, 100 μg ml^-1^, and 70 μg ml^-1^, respectively.

**Table 1:** *S. mutans* strains and plasmids used in this study.

Strain and plasmid	Relevant characteristics	Reference
***S. mutans***		
XC	Wild type	Koga et al. (1989)
Δ*gdpP*-Int	*S. mutans* XC containing the IFDC2 cassette in place of *gdpP*	This study
Δ*gdpP*	*S. mutans* XC lacking *gdpP* without antibiotic cassettes	This study
Δ*dhhP*-Int	*S. mutans* XC containing t the IFDC2 cassette in place of *dhhP*	This study
Δ*dhhP*	*S. mutans* XC lacking *dhhP* without antibiotic cassettes	This study
Δ*gdpP*Δ*dhhP* -Int	*S. mutans*Δ*gdpP* containing t the IFDC2 cassette in place of *dhhP*	This study
Δ*gdpP*Δ*dhhP*	*S. mutans* XC lacking *gdpP* and *dhhP* without antibiotic cassettes	This study
Δ*gdpP*-com	*S. mutans*Δ*gdpP* containing pSM2140	This study
Δ*dhhP*-com	*S. mutans*Δ*dhhP* containing pSM1297	This study
**Plasmid**		
pMCL200	Cm^R^, cloning vector	Nakano et al. (1995)
pKOgdpP-Int	Cm^R^ and Em^R^; pMCL200 derivative containing the IFDC2 cassette flanked by the upstream and downstream regions of *gdpP*	This study
pKOgdpP	Cm^R^; pMCL200 derivative containing the upstream and downstream regions of *gdpP*	This study
pKOdhhP-Int	Cm^R^ and Em^R^; pMCL200 derivative containing the IFDC2 cassette flanked by the upstream and downstream regions of *dhhP*	This study
pKOdhhP	Cm^R^; pMCL200 derivative containing the upstream and downstream regions of *dhhP*	This study
pCold ProS2	Ap^R^; GST fusion expression vector	Takara Bio
pGdpP- ProS2	Ap^R^; Cold ProS2 derivative containing *gdpP*	This study
pDhhP- ProS2	Ap^R^; Cold ProS2 derivative containing *dhhP*	This study
pJY	Km^R^; a shuttle vector between *Streptococcus* and *Escherichia*	Yang et al. (2009)
pSM2140	Km^R^; pJY derivative containing *gdpP*	This study
pSM1297	Km^R^; pJY derivative containing *dhhP*	This study

### Preparation of Recombinant GdpP and DhhP Proteins

Truncated *gdpP* and intact *dhhP* genes were amplified by PCR using primers listed in **Supplementary Table S1** and *S. mutans* genomic DNA as a template, and cloned into the pCold ProS2 vector (Takara Bio, Otsu, Japan). The N-terminal region (108 amino acids), which were not contained in the recombinant GdpP, was determined as previously described (Bai et al., 2013). The resulting plasmids (**Table 1**) were verified by DNA sequencing. *E. coli* BL21 cells harboring either plasmid were grown at 37°C to absorbance at 595 nm (*A*_595_) of ∼0.5. The bacterial culture was then cooled to 16°C and isopropyl-β-D-thiogalactoside was added to a final concentration of 0.3 mM. After induction at 16°C for 24 h, cells were sonicated and cell debris was removed by centrifugation at 50,000 × *g* and 4°C for 1 h. The supernatant was loaded onto a Co-based TALON column (Takara Bio, Otsu, Japan) pre-equilibrated with TBS (Tris-HCl buffer, pH 7.5, supplemented with 300 mM NaCl). After the resin was extensively washed with TBS containing 10 mM imidazole, proteins were eluted using 250 mM imidazole in TBS. Each eluate was dialyzed against 10 mM Tris-HCl (pH 7.4) and stored at -20°C following addition of an equal volume of 80% glycerol. The protein concentration was measured using a Pierce BCA Protein Assay Kit (Thermo Scientific, Tokyo, Japan), and the purity of samples was analyzed by sodium dodecyl sulfate-polyacrylamide gel electrophoresis (SDS-PAGE).

### Liquid Chromatography Coupled With Tandem Mass Spectrometry (LC-MS/MS)

Adenosine monophosphate (AMP), pApA, c-di-AMP, and c-di-GMP were detected in reaction mixtures using a Nexera2 LC system (Shimadzu, Kyoto, Japan) connected to a triple quadrupole mass spectrometer (LC-MS 8040; Shimadzu) equipped with an electrospray ionization (ESI) source and operated in positive ion mode for pApA and negative ion mode for AMP, c-di-AMP, and c-di-GMP. Samples were separated using a reversed-phase Mastro SP column (2.1 × 100 mm; Shimadzu). The mobile phases were as follows: (A) 10 mM ammonium acetate/acetonitrile (90:10 v/v) and (B) 50 mM ammonium acetate:acetonitrile (80:20 v/v). A linear gradient from 0 to 60% solvent B over 9 min was applied at a flow rate of 0.5 ml min^-1^. The *A*_254_ value was monitored to quantify AMP, pApA, and c-di-AMP in reaction mixtures. ESI multiple reaction monitoring (MRM) mode was used to identify the compounds. The temperature of the ESI capillary was maintained at 300°C. The drying gas was introduced at a temperature of 300°C and a flow rate of 4 l min^-1^. The nebulizer gas pressure was 10 psi. Each sample (1 μl) was injected into the LC capillary. All solvents used in these experiments were LC-MS grade from Wako Pure Chemicals (Osaka, Japan) or Sigma-Aldrich Japan.

### Enzyme Reactions

To determine kinetic parameters, reaction mixtures (50 μl) contained 50 mM potassium phosphate buffer (pH 5.0 for recombinant GdpP and pH 7.0 for recombinant DhhP), 1 mM MnCl_2_, 100 μM c-di-AMP, pApA, or c-di-GMP, and 1.2 μg of truncated recombinant GdpP ml^-1^ or 0.36 μg of recombinant DhhP ml^-1^. After 10 min, reactions were terminated by addition of 50 μl TE (50 mM Tris-HCl pH 8.0, 10 mM EDTA) -saturated phenol, followed by addition of the same volume of chloroform. After dilution with distilled water, the aqueous phase was analyzed by LC-MS/MS. Reaction mixtures at various concentrations of substrates (0–0.3 mM) were assayed to determine the kinetic properties of the enzymes. The assay mixtures used for divalent metal ion screening contained 1 mM MgCl_2_, 1 mM ZnCl_2_, 1 mM CoCl_2_, 1 mM CaCl_2_, or different concentrations of MnCl_2_ (0.1, 1, 5, or 10 mM). To analyze the optimal pH, reaction mixtures consisted of 50 mM potassium phosphate buffer at pH values adjusted to 4.0, 5.0, 6.0, 7.0, 8.0, or 9.0. To investigate the optimal reaction temperature, reaction mixtures were incubated at 20, 30, 37, 40, 50, or 60°C.

### Construction of Deletion and Complemented *Streptococcus mutans* Strains

Markerless mutagenesis was performed to construct three in-frame *S. mutan*s XC deletion mutants, namely, Δ*gdpP*, Δ*dhhP*, and Δ*gdpP*Δ*dhhP* (**Table 1** and **Supplementary Figures S1, S2, S3**). A two-step transformation protocol using an IFDC2 cassette was used as described previously (Xie et al., 2011). This cassette contains an erythromycin resistance gene and a gene encoding mutated phenylalanine tRNA synthetase that incorporates the toxic *p*-Cl-Phe into proteins during translation. Introduction of the IFDC2 cassette altered the phenotype of the wild-type (WT) strain from *p*-Cl-Phe resistance and erythromycin sensitivity to *p*-Cl-Phe sensitivity and erythromycin resistance. First, three DNA fragments (an upstream targeting sequence, the IFDC2 cassette, and downstream targeting sequence) were individually generated by PCR, ligated using an In-fusion HD Cloning kit (Takara Bio, Otsu, Japan), and inserted into the cloning vector pMCL200 (Nakano et al., 1995). *S. mutans* XC cells were transformed with the respective linearized plasmids as previously described (Yoshida et al., 2005). The sensitivity of transformants (intermediate strains Δ*gdpP*-Int and Δ*dhhP*-Int) to *p*-Cl-Phe was then verified (**Supplementary Figures S1, S2, S3**). In the second step, two DNA fragments (an upstream targeting sequence and a downstream targeting sequence) were linked using an In-fusion HD Cloning kit and then subcloned into pMCL200. The linearized plasmids were used to transform Δ*gdpP*-Int and Δ*dhhP*-Int strains. The transformants were selected and patched onto fresh BHI plates containing 200 μg *p*-Cl-Phe ml^-1^, and onto BHI plates containing 20 μg erythromycin ml^-1^, to confirm loss of the IFDC2 cassette. Deletion of the *gdpP* and *dhhP* genes in the mutant strains, designated Δ*gdpP* and Δ*dhhP*, respectively, was confirmed by PCR and DNA sequencing. Similarly, a double mutant strain, Δ*gdpP*Δ*dhhP* was constructed in the Δ*gdpP* background using the two-step transformation protocol, as described above (**Supplementary Figure S3**). All strains and plasmids used are listed in **Table 1**.

To facilitate genetic complementation studies of mutants of *S. mutans*, shuttle plasmids were constructed using pJY (Yang et al., 2009) with a constitutive promoter (CP25) (Hansen et al., 2001). After PCR-amplified *gdpP* and *dhhP* genes with a Shine-Dalgarno sequence (Shine and Dalgarno, 1974) were inserted into the *Bam*HI and *Nco*I sites of pJY using an In-fusion HD Cloning kit (Takara Bio, Otsu, Japan), the integrity of the cloned sequence was verified by DNA sequencing. The resulting plasmids, pSM2140 and pSM1297 (**Table 1**), were used to transform *S. mutans*Δ*gdpP* and Δ*dhhP*, respectively, to construct Δ*gdpP-*com and Δ*dhhP-*com (**Table 1**).

### Biofilm Assay

Biofilm formation assays were performed on polystyrene plates as previously described (Yano et al., 2012) with several minor modifications. Bacterial cultures used for biofilm formation were prepared by inoculating overnight cultures of *S. mutans* strains into fresh BHI broth at a ratio of 1:20, and anaerobically incubated at 37°C for 24 h. Culture aliquots (5 μl) were inoculated into 95 μl of fresh BHI broth in a 96-well flat-bottomed polystyrene plate. To evaluate the effect of sucrose on biofilm formation, 1.0% sucrose was added to the media. After incubation at 37°C for 24 h, bacterial cultures were gently discarded, and plates were carefully washed three times with phosphate-buffered saline (PBS) to remove non-adhered cells and then stained with 0.2% (w/v) Victoria Blue for 15 min. Bound dye was dissolved using 100 μl of 99% methanol, and biofilm volume was evaluated by measuring the absorbance at 595 nm (*A*_595_). When *A*_595_ values were >2.0, they were measured again after appropriate dilution. A blank value (medium alone) was subtracted from each value. Data are represented as mean ± standard deviation from 12 replicates in three independent experiments.

### Morphological Analyses

To observe planktonic *S. mutans* cells, cultures were grown for 24 h, inoculated into fresh BHI broth at a ratio of 1:20, and incubated until the *A*_595_ value was ∼0.3 (mid-log phase). Aliquots of streptococcal cultures (5 μl) were fixed and stained using a conventional Gram staining method for optical microscopy. Biofilm formation after 12 h was also assessed using scanning electron microscopy (SEM) as previously described (Konno et al., 2015).

### Statistical Analysis

Data were analyzed by one-way analysis of variance (ANOVA) followed by Tukey’s *post hoc* tests at a significance level of *p* < 0.01.

## Results

### Identification and Purification of Putative c-di-AMP Phosphodiesterases GdpP and DhhP

**FIGURE 1:**
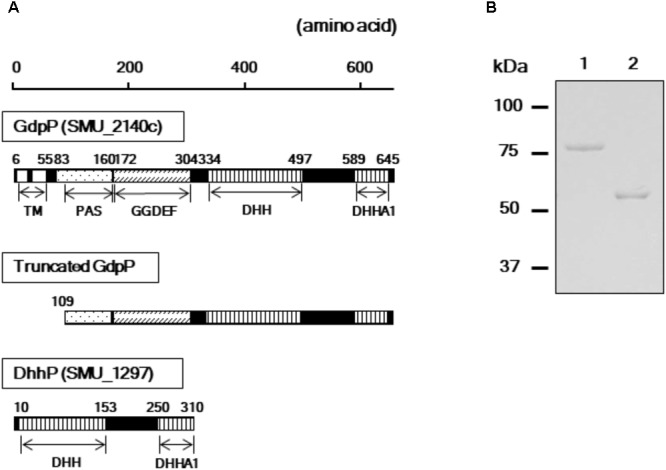
Purification of rGdpP_109-657_-ProS2 and rDhhP-ProS2. **(A)** Protein domain architecture. Numbers indicate amino acid positions in the full-length protein sequences. The N-terminus of the intact GdpP (657 amino acids) contains two transmembrane helices (TMs), a PAS domain, a GGDEF domain, a DHH domain, and a DHHA1 domain. rGdpP_109-657_-ProS2 generated in this study does not contain a TM. Both rGdpP_109-657_-ProS2 and rDhhP-ProS2 were generated with N-terminal 23 kDa ProS2 domains to increase their stability. **(B)** Sodium dodecyl sulfate-polyacrylamide gel electrophoresis analysis. Lane 1, rGdpP_109-657_-ProS2; Lane 2, rDhhP-ProS2. The positions of the molecular mass markers are shown on the left.

A homology search revealed that the amino acid sequences of GdpP (SMU_2140c) and DhhP (SMU_1297) from *S. mutans* are 56 and 68% identical with Pde1 and Pde2 from *S. pneumoniae*, respectively, and several functional domains are shared between the respective homologs (**Figure 1A**). To evaluate the enzymatic functions of GdpP and DhhP in *S. mutans*, we attempted to purify the recombinant proteins following heterologous expression in *E. coli* but failed to obtain intact GdpP. Thus, genes encoding a truncated GdpP variant lacking transmembrane helices and a part of PAS domain (spanning amino acids 1–108), and an intact DhhP were cloned, and recombinant proteins were purified from *E. coli* lysates (**Figure 1**). The N-terminal hydrophobic transmembrane domains are dispensable for c-di-AMP phosphodiesterase activity (Corrigan et al., 2011; Rao et al., 2011; Bai et al., 2013). In addition, the PAS domain also did not affect the enzymatic function of GdpP (Bai et al., 2013). Each protein was generated as a fusion construct with a 23 kDa ProS2 tag that rendered the recombinant protein soluble and stable. The recombinant GdpP and DhhP were designated rGdpP_109-657_-ProS2 and rDhhP-ProS2, respectively. The molecular masses of the denatured fusion proteins were consistent with their predicted molecular masses (i.e., 84 kDa and 57 kDa for rGdpP_109-657_-ProS2 and rDhhP-ProS2, respectively; **Figure 1B**).

### Enzymatic Characterization of rGdpP_109-657_-ProS2 and rDhhP-ProS2 From *S. mutans* as c-di-AMP Phosphodiesterases

**FIGURE 2:**
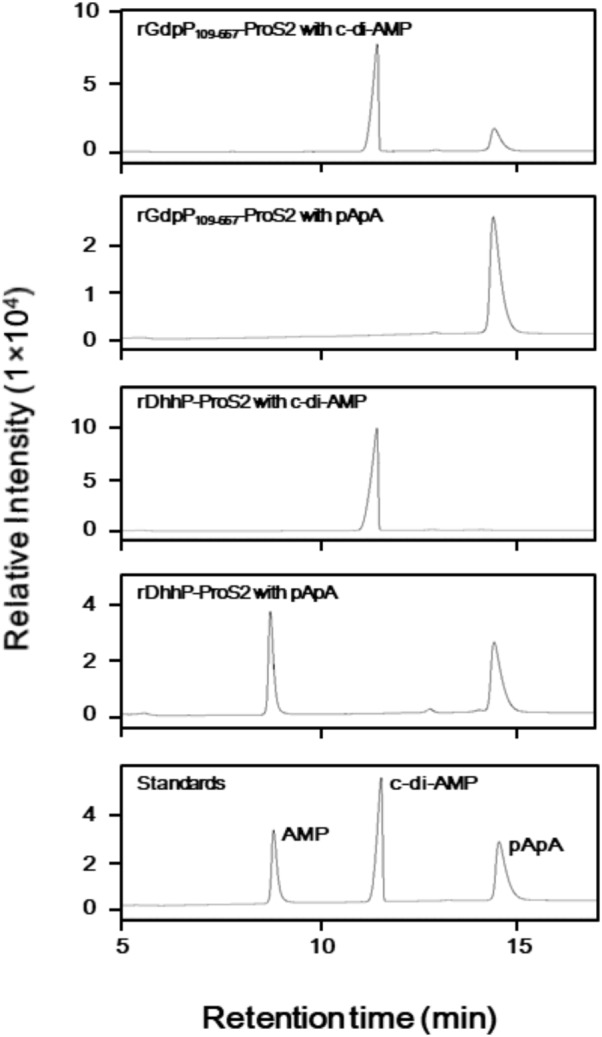
Phosphodiesterase activity of rGdpP_109-657_-ProS2 and rDhhP-ProS2 by liquid chromatography (LC) analysis. Reaction mixtures (50 μl) contained 50 mM potassium phosphate buffer (pH 5.0 for GdpP, pH 7.0 for rDhhP-ProS2), 1 mM MnCl_2_, 100 μM substrate (c-di-AMP or pApA), and 1.2 μg ml^-1^ rGdpP_109-657_-ProS2 or 0.36 μg ml^-1^ rDhhP-ProS2. Reactions were incubated for 15 min and terminated by addition of TE-saturated phenol and chloroform, and an aliquot (1 μl) of the aqueous phase was injected onto the LC column. Each sample was separated by LC and monitored at 254 nm. Purified AMP, c-di-AMP, and pApA were used as standards.

LC analysis of *A*_254_ values revealed that incubation of rGdpP_109-657_-ProS2 with c-di-AMP resulted in the production of pApA (**Figure 2**), and this was further confirmed by MRM mode LC-MS/MS, in which transitions from *m*/*z* 677.4 (1+, precursor ion) to *m*/*z* 136.1 (product ion) and *m*/*z* 97.0 (product ion) were observed. However, pApA was not degraded by rGdpP_109-657_-ProS2, but incubation of rDhhP-ProS2 with pApA resulted in the production of AMP, and this was also confirmed by LC-MS/MS, in which the transition of *m*/*z* 346.05 (1-, precursor ion) to *m*/*z* 79.1 (product ion) was observed. Unlike Pde2 from *S. pneumoniae*, c-di-AMP was not cleaved by rDhhP-ProS2 (**Figure 2**), even when a large amount of rDhhP-ProS2 (100-fold more than usual) was incubated with c-di-AMP for a long incubation time (12-fold longer than usual). This suggests that direct production of AMP from c-di-AMP by DhhP is negligible in *S. mutans.* Neither rGdpP_109-657_-ProS2 nor rDhhP-ProS2 cleaved c-di-GMP (data not shown), indicating that c-di-GMP is not a substrate for either of GdpP and DhhP.

**FIGURE 3:**
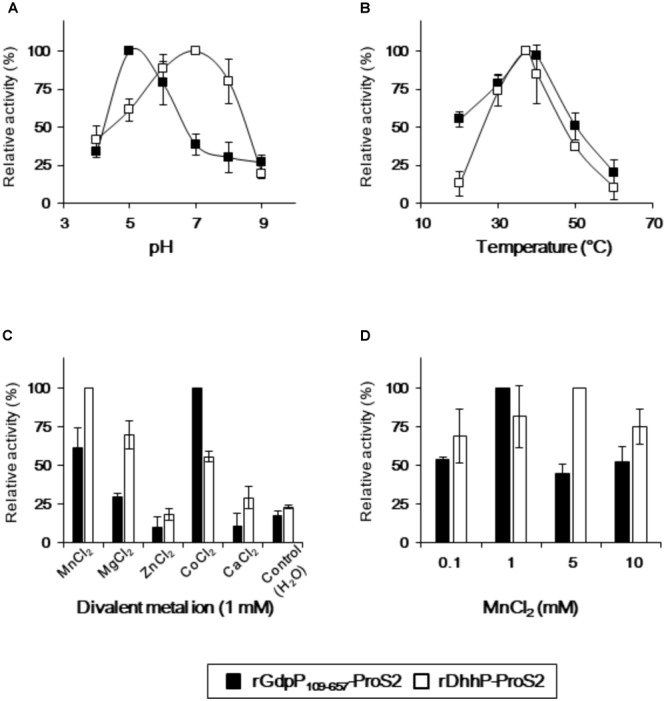
Optimization of reaction conditions for rGdpP_109-657_-ProS2 and rDhhP-ProS2. The relative activity was measured at different **(A)** pH values, **(B)** temperatures, **(C)** divalent metal ion concentrations, and **(D)** MnCl_2_ concentrations. Enzyme activity is presented relative to the maximum activity for each variable. Data are presented as mean ± standard deviation from three independent experiments.

Reaction conditions for rGdpP_109-657_-ProS2 and rDhhP-ProS2 from *S. mutans* were optimized by quantifying pApA and AMP produced from c-di-AMP and pApA, respectively, in different pH, temperatures, and divalent ions (**Figure 3**). Maximum activity of rGdpP_109-657_-ProS2 was observed at pH 5.0 and 37°C, and Co^2+^ was the most effective of the divalent metal ions tested. Mn^2+^ also stimulated the enzyme with an optimal concentration of 1 mM, equivalent to ∼60% of the activity with Co^2+^. Interestingly, the enzymatic activity of rGdpP_109-657_-ProS2 in the presence of Zn^2+^ or Ca^2+^ was lower than that of the control, suggesting that these ions inhibit catalytic activity. rDhhP-ProS2 exhibited highest activity under slightly different conditions, with an optimal pH of 7.0 and an optimal temperature of 37°C. Of the divalent metal ions tested, Mn^2+^ was the most effective for stimulating enzymatic activity, and rDhhP-ProS2 was also stimulated by Mg^2+^, Co^2+^, and Ca^2+^, and inhibited by Zn^2+^, but not by Ca^2+^.

Kinetic parameters of rGdpP_109-657_-ProS2 and rDhhP-ProS2 enzymes were determined using the initial rate method, with end products (separated by LC) quantified using *A*_254_ values (**Table 2**).

**Table 2:** Kinetic properties for the reactions catalyzed by rGdpP_109-657_-ProS2 and rDhhP-ProS2 from *S. mutans*.

Enzyme	Substrate	End product	*K*_m_ (μM)	*V*_max_ (μmol mg^-1^ min^-1^)	*k*_cat_ (sec^-1^)
rGdpP_109-657_-ProS2	c-di-AMP	pApA	89.7 ± 7.29	4.58 ± 0.23	4.02 ± 0.20
rDhhP-ProS2	pApA	AMP	199 ± 65.4	32.1 ± 7.08	36.5 ± 8.08

### Deletion of *dhhP* in *S. mutans* Results in Suppression of Growth

**FIGURE 4:**
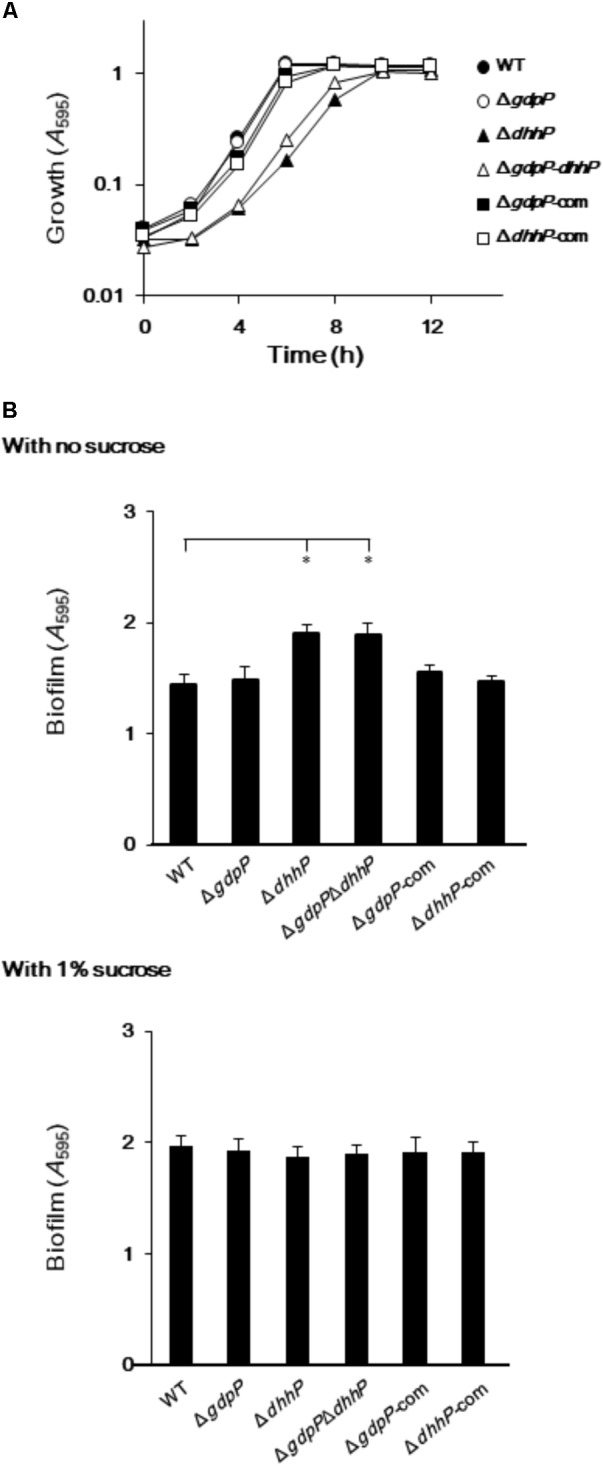
Growth and biofilm formation of *Streptococcus mutans* XC, Δ*gdpP*, Δ*dhhP*, Δ*gdpP*Δ*dhhP*, Δ*gdpP*-com, and Δ*dhhP-*com strains. **(A)** *A*_595_ values of bacterial cell cultures, monitored every 2 h. **(B)** Sucrose-independent and -dependent biofilm formation in 96-well polystyrene plates after 24 h. Data are expressed as the mean ± standard deviation from 12 replicates from three independent experiments. Data were analyzed by one-way analysis of variance (ANOVA) followed by Tukey’s *post hoc* tests. ^∗^*p* < 0.01.

To examine the biological roles of GdpP and DhhP, three markerless in-frame mutant strains, in which the potentially confounding polar effect of the promoter of the antibiotic resistance gene on the downstream genes was eliminated, were constructed (**Supplementary Figures S1, S2, S3**). In addition, strains complemented with *gdpP* and *dhhP* were also constructed (**Table 1**). Since c-di-AMP homeostasis plays an important role in cell growth in some bacteria (Mehne et al., 2013; Ye et al., 2014), the growth of *S. mutans* and its derivatives was examined by measuring *A*_595_ values (**Figure 4A**). The growth of the Δ*gdpP* strain was similar to that of the WT strain, while the growth of Δ*dhhP* and Δ*gdpP*Δ*dhhP* strains was slower than that of the WT. Furthermore, the slow growth of the *ΔdhhP* strain was restored by complementation with *dhhP* (**Figure 4A**).

### Deletion of *dhhP* in *S. mutans* Results in Increased Biofilm Production

When *S. mutans* WT and derivative strains were incubated for 24 h in the absence of sucrose, biofilm formation was significantly more pronounced in strains Δ*dhhP* and Δ*gdpP*Δ*dhhP* than in the other strains (**Figure 4B**). By contrast, the amount of biofilm formed by the Δ*gdpP* mutant in the absence of sucrose was not significantly different from that of the WT strain when incubated for 24 h (**Figure 4B**). Importantly, the amount of biofilm produced by the Δ*dhhP* strain was restored to WT levels upon complementation with *dhhP* (**Figure 4B**). These findings revealed that the function of DhpP plays an important role in stimulating biofilm formation. Biofilm formation was not significantly different among strains in the presence of 1.0% sucrose (**Figure 4B**).

**FIGURE 5:**
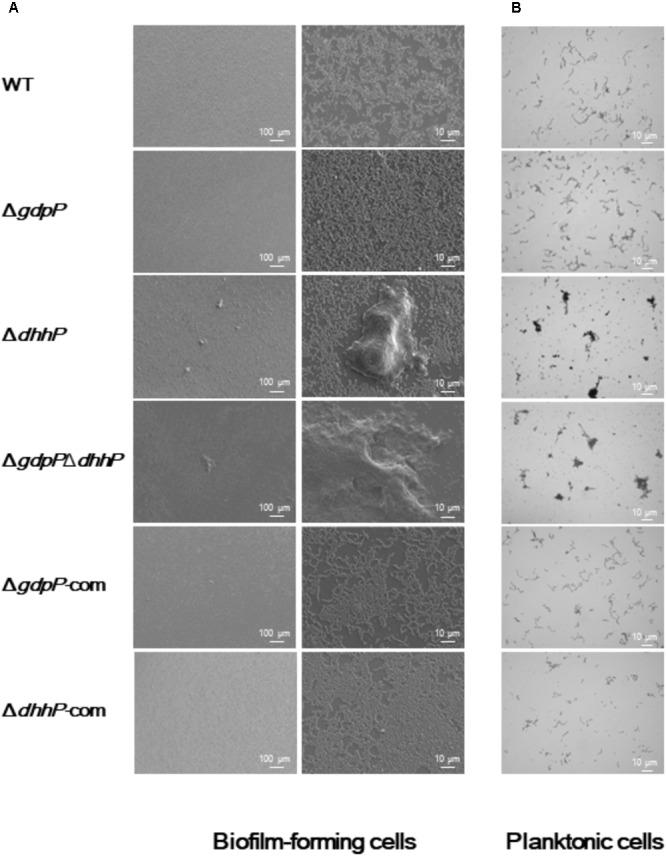
Morphology of *S. mutans* XC, Δ*gdpP*, Δ*dhhP*, Δ*gdpP*Δ*dhhP*, Δ*gdpP*-com, and Δ*dhhP-*com strains. **(A)** Scanning electron microscopy images of biofilms formed after 12 h on a cover glass. Each image is representative of 3–5 independent experiments. **(B)** Optical microscopy analysis of *S. mutans* strains grown in liquid media. Cells grown to log phase were observed after conventional Gram staining. Each image is representative of three independent experiments.

Optical and electron microscopy observations showed that cells of both Δ*dhhP* and Δ*gdpP*Δ*dhhP* strains clumped together to form large bacterial aggregates of ∼0.1 mm in diameter under both biofilm and planktonic conditions, whereas cell clumps were not observed in cultures of WT, Δ*gdpP*, Δ*gdpP-*com, or Δ*dhhP-*com cells (**Figure 5**), revealing that lack of DhhP resulted in the formation of *S. mutans* aggregates.

## Discussion

The secondary messenger c-di-AMP was recently identified in bacteria and appears to be required for cell viability (Song et al., 2005; Woodward et al., 2010; Corrigan et al., 2011; Luo and Helmann, 2012; Mehne et al., 2013). c-di-AMP is synthesized by most of Gram-positive bacteria (Woodward et al., 2010; Corrigan et al., 2011; Kamegaya et al., 2011; Bai et al., 2013; Du et al., 2014), whereas the molecule is also present in some Gram-negative pathogens (Barker et al., 2013) and Spirochaetae (Ye et al., 2014). By contrast, the enzymes associated with c-di-GMP and its functions for the secondary messenger have been investigated in a diverse range of bacterial species (Jenal et al., 2017).

GdpP and DhhP in *S. mutans* are orthologs of Pde1 and Pde2 in *S. pneumoniae*, respectively, despite sharing relatively low sequence identity. Homology analyses revealed that streptococcal species, including *S. sobrinus, S. gordonii, S. salivarius, S. pyogenes, S. agalactiae, S. oralis, S. suis*, and *S. anginosus*, contain one ortholog of GdpP and one ortholog of DhhP. Thus, the two distinct c-di-AMP phosphodiesterases characterized in this study appear to be present in most streptococci.

The rGdpP_109-657_-ProS2 appears to share the same substrate specificity as the Pde1 enzyme from *S. pneumoniae* (Bai et al., 2013). By contrast, the substrate specificity of rDhhP-ProS2 was different from that of Pde2 from *S. pneumoniae*, which produced AMP not from c-di-AMP but from pApA (**Figure 2**), whereas Pde2 from *S. pneumoniae* produced AMP both from c-di-AMP and pApA. Notably, the enzymatic activity of *S. pneumoniae* Pde2 to produce AMP from c-di-AMP was much lower (approximately 1/2000) than that of the same enzyme to produce AMP from pApA (Bai et al., 2013). Based on these previous findings and our current results (**Figure 2**), we conclude that DhhP has no detectable capacity to degrade c-di-AMP to produce AMP, and mainly cleaves pApA to AMP in *S. mutans*. It is probable that in *S. mutans*, c-di-AMP is degraded to the pApA intermediate by GdpP, and this is subsequently degraded to AMP by DhhP.

The optimal pH for the enzymatic activities of rGdpP_109-657_-ProS2 and rDhhP-ProS2 (pH 5.0 and 7.0, respectively) was lower than that of the orthologs listed above (mostly pH 8.0 or 8.5). This lower optimal pH for rGdpP_109-657_-ProS2 and rDhhP-ProS2 might be related to the ability of *S. mutans* to survive in more acidic environments (Hamilton and Buckley, 1991). DHH family proteins generally require a divalent cation for function (Yamagata et al., 2002). Indeed, Mn^2+^ is indispensable for the enzymatic activity of many phosphodiesterases that act on c-di-AMP, including Pde2 from *S. pneumoniae* (Bai et al., 2013), CnpB from *Mycobacterium tuberculosis* (Yang et al., 2014), DhhP from *B. burgdorferi* (Ye et al., 2014), and GdpP from *B. subtilis* (Rao et al., 2011). These findings, together with our current results (**Figure 3C**), suggest that Mn^2+^ might be the physiological metal ion of GdpP and DhhP proteins. Interestingly, the enzymatic activity of these enzymes was inhibited by Zn^2+^ and Ca^2+^, consistent with previous reports of inhibition of phosphodiesterase activity by Zn^2+^ and Ca^2+^ in several bacteria (Rao et al., 2011; Ye et al., 2014; Tang et al., 2015).

Biofilm formation by Δ*gdpP* was not significantly different from that of the WT strain (**Figure 4**), unlike other streptococcal species; deletion of *gdpP* resulted in increased biofilm production by *S. suis* (Du et al., 2014) and *S. pneumoniae* (Bai et al., 2013). Our data from three independently constructed mutant strains (Δ*dhhP*, Δ*gdpP*Δ*dhhP*, and Δ*dhhP*-com) clearly demonstrated that deletion of the *dhhP* gene led to increased biofilm formation, probably due to aggregation of *S. mutans* (**Figures 4B, 5**). Bacterial aggregates of Δ*dhhP* and Δ*gdpP*Δ*dhhP* strains could act as scaffolds on which thick biofilms are formed. However, the exact mechanism by which deletion of the *dhhP* gene is associated with the formation of cell clumps remains to be elucidated. Given the products of the rDhhP-ProS2 enzymatic reaction (**Figure 2**), exhaustion of AMP in bacterial cells and/or accumulation of pApA might affect biofilm formation. Otherwise, it would be possible that AMP has an inhibitory effect on biofilm formation. Deletion of *gdpP*, which leads to exhaustion of AMP in *S. mutans* cells, did not result in aggregation of the bacteria (**Figure 5**), suggesting that the concentration of AMP in cells might not be associated with formation of biofilms. Further studies are necessary to precisely elucidate the relationship between increased biofilm formation and deletion of *dhhP* since the intercellular level of c-di-AMP affects the expression of ∼700 genes in *B. subtilis* (Gundlach et al., 2016). Peng et al. (2016) reported that biofilm formation by a *gdpP*-deficient mutant of *S. mutans* UA159 was greater than the WT strain in the presence of sucrose. Contrary to this previous report, we showed that the amount of biofilm formed by *S. mutans* XC and its derivatives in the presence of 1.0% sucrose, which was higher than that in the absence of sucrose, was not significantly different among the strains tested (**Figure 4B**). *S. mutans* glucosyltransferases, including GTF-I and GTF-SI, synthesize water-insoluble glucans from sucrose (Kuramitsu, 1993), thereby forming extraordinarily strong and thick biofilms (Bowen and Koo, 2011). Therefore, it might be difficult to precisely evaluate how *gdpP* and *dhhP* deletion affects biofilm formation because large amounts of insoluble glucans produced in the presence of sucrose could mask the relatively weak effect of *gdpP* and *dhhP* deletion on biofilm formation.

Growth as a biofilm is usually triggered to assist survival in inhospitable conditions, and deletion of the *dhhP* gene may constitute a stressful situation for bacteria. Interestingly, the DhhP protein identified as a phosphodiesterase in the current study was previously reported to be essential for the superoxide stress response (Zhang and Biswas, 2009). Together with the existing literature, our present findings indicate that DhhP protein and related molecules such as c-di-AMP, pApA, and AMP might play an important role in the response of *S. mutans* to stress conditions.

## Conclusion

Herein, two *S. mutans* c-di-AMP phosphodiesterases, GdpP, and DhhP, were identified and characterized. rGdpP_109-657_-ProS2 degrades c-di-AMP to produce pApA, which is subsequently degraded by rDhhP-ProS2 to yield AMP. Deletion of *dhhP* in *S. mutans* resulted in the formation of large cell clumps, leading to increased biofilm formation.

## Author Contributions

YY conceived of and designed the experiments. HK and YY acquired the data. HK, YY, and KN analyzed the data. HK, YY, KN, JT, and YH interpreted the data.

## Conflict of Interest Statement

The authors declare that the research was conducted in the absence of any commercial or financial relationships that could be construed as a potential conflict of interest.
