# Supplementary material for: Biological and Biochemical Roles of Two Distinct Cyclic Dimeric Adenosine 3′,5′-Monophosphate- Associated Phosphodiesterases in Streptococcus mutans
Source: Front Microbiol. 2018 Sep 27;9:2347. doi: 10.3389/fmicb.2018.02347 (PMC6170606; doi:10.3389/fmicb.2018.02347)
Supplement: Supplementary file 1 [file Data_Sheet_1.PDF]

Supplemental TABLE S1. Oligonucleotide primers used in this study

Purpose	Designation	Primer	Sequence (5' to 3') *
Construction of recombinant protein			
	GdpP	121914-truncated-F-Bam	**AAGGATCC**ACGAAAGAAGATGGTGAATTTG
		012814-2140-R-Sal	**AAGTCGAC**TTAAGCTTCCTCGTTTCCTG
	DhhP	040714-1297-F-Bam	**AAGGATCC**ACTGCTTTTAAAACTATTCTAGCTAAAAT
		040714-1297-R-Sal	**AAGTCGAC**TTTTAGCAGATTTTTTAATTCCTGA
Construction of mutant strain			
	IFDC2 cassette	IFDC2-F	CCGAGCAACAATAACACTC
		IFDC2-R	GAAGCTGTCAGTAGTATACC
	Upstream region for Δ*gdpP*-Int	102615-2140ko-F1-Infu	CTCTAGAACTAGTGGATCCACCTTATCGTTTACTAACATCGC
		090715-2140KO-R1-IFDC2	GAGTGTTATTGTTGCTCGGTTAACAGTGGTGAATCTGGAA
	Downstream region for Δ*gdpP*-Int	090715-2140ko-F2-IFDC2	GGTATACTACTGACAGCTTCAAGCCCGCAAACTCTTATTAG

	102615-2140kO-R2-Infu	CCCCCCCTCGAGGTCGACACGATGAGCATATTTATAAAAATCTTC
Upstream region for Δ*gdpP*	102615-2140ko-F1-Infu	CTCTAGAACTAGTGGATCCACCTTATCGTTTACTAACATCGC
	090715-2140ko2-R2	ATAACTTTCATCTTATATCTAAACCTCTTAAGCCCTAATTATATC
Downstream region for Δ*gdpP*	090715-2140ko2-F2	TAAGAGGTTTAGATATAAGATGAAAGTTATTTTTTTAGCAGAT
	102615-2140kO-R2-Infu	CCCCCCCTCGAGGTCGACACGATGAGCATATTTATAAAAATCTTC
Upstream region for Δ*dhhP*-Int	100615-1297-F1-InFu	CTCTAGAACTAGTGGATCCTTCTGATTTTGTTGCCATTAAC
	090715-1297KO-R1-IFDC2	GAGTGTTATTGTTGCTCGGACCTGACTGCCTAATGCGTCAGGGTCTG
Downstream region for Δ*dhhP*-Int	090715-1297KO-F2-IFDC2	GGTATACTACTGACAGCTTCCGGCCATCCATTAGCAAGTGGTGC
	100615-1297-R2-InFu	CCCCCCCTCGAGGTCGACTGATGGACAAATTGCCAGT

	Upstream region for Δ*dhhP*	100615-1297-F1-InFu	CTCTAGAACTAGTGGATCCTTCTGATTTTGTTGCCATTAAC
		090715-1297ko2-R2	AGTTTTTTATACTAAAATTCCTCAATTCATTTTTTTACA
	Downstream region for Δ*dhhP*	090715-1297ko2-F2	TTGAGGAATTTTAGTATAAAAAACTTGTCAGTCGTCA
		100615-1297-R2-InFu	CCCCCCCTCGAGGTCGACTGATGGACAAATTGCCAGT
Construction of complemented strains			
	gdpP	022618-RBS-2140-Bam	ATCTGCGGCCGCCTAGGATCC-TAGGGCTTAAGAGGTTTAGATAATGAAAA
		022618-2140-Nco	CCGGAGAATTCCATGG-TTAAGCTTCCTCGTTTCCTGTAAA
	dhhP	022618-RBS-1297-Bam	ATCTGCGGCCGCCTAGGATCC-TGAATTGAGGAATTTATGACTGCTTTTAA
		022618-1297-Nco	CCGGAGAATTCCATGG-CTATTTTAGCAGATTTTTTAATTCCTGATAAATG

* Bold nucleotides indicate restriction endonuclease sites incorporated to facilitate cloning. Underlined nucleotides indicate an overlapping region of the IFDC2 cassette, pMCL200, or pJY.
