# Supplementary material for: Biological and Biochemical Roles of Two Distinct Cyclic Dimeric Adenosine 3′,5′-Monophosphate- Associated Phosphodiesterases in Streptococcus mutans
Source: Front Microbiol. 2018 Sep 27;9:2347. doi: 10.3389/fmicb.2018.02347 (PMC6170606; doi:10.3389/fmicb.2018.02347)
Supplement: Supplementary file 2 [file Data_Sheet_2.PDF]

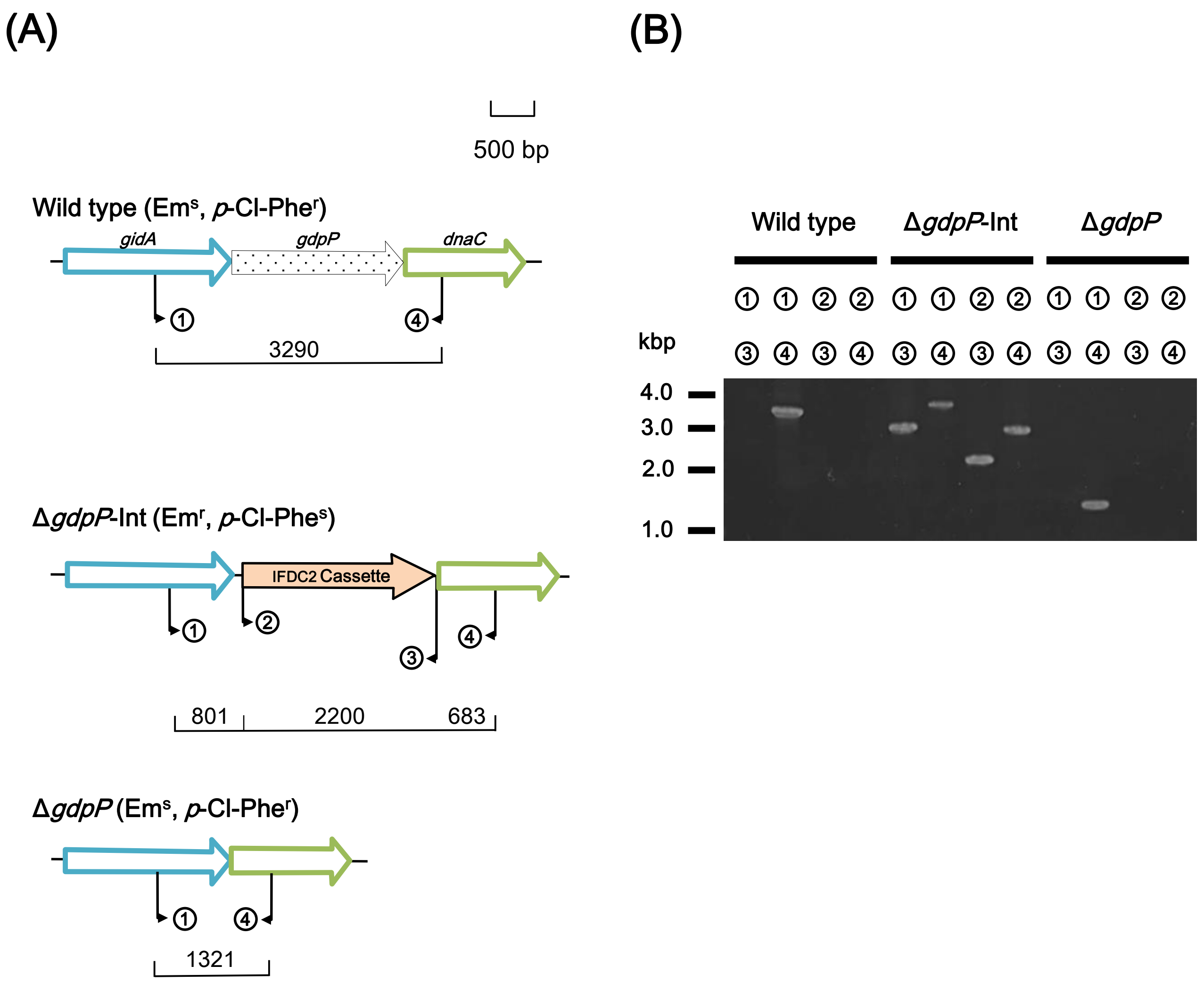

Supplemental Figure S1. Construction of a markerless mutant of *S. mutans* XC lacking *gdpP*. Chromosomal gene arrangement **(A)** and verifying PCR analyses **(B)** are shown . The *gdpP* gene was initially replaced by the IFDC2 cassette of pKOgdpP-Int, yielding an intermediate mutant strain, Δ*gdpP*-Int. The resulting strain was selected using erythromycin. Subsequently, the IFDC2 cassette was replaced by a linear construct containing two linked homologous fragments without the selection cassette in pKOgdpP. The transformants were then selected on plates containing *p*-Cl-Phe. The resulting markerless mutant (Δ*gdpP*) is sensitive to erythromycin and resistant to *p*-Cl-Phe. Each circled number indicates a PCR primer used for mutant verification. The distances between primers are given in bp. Each DNA fragment was PCR-amplified using the indicated primers. DNA size standards are shown .

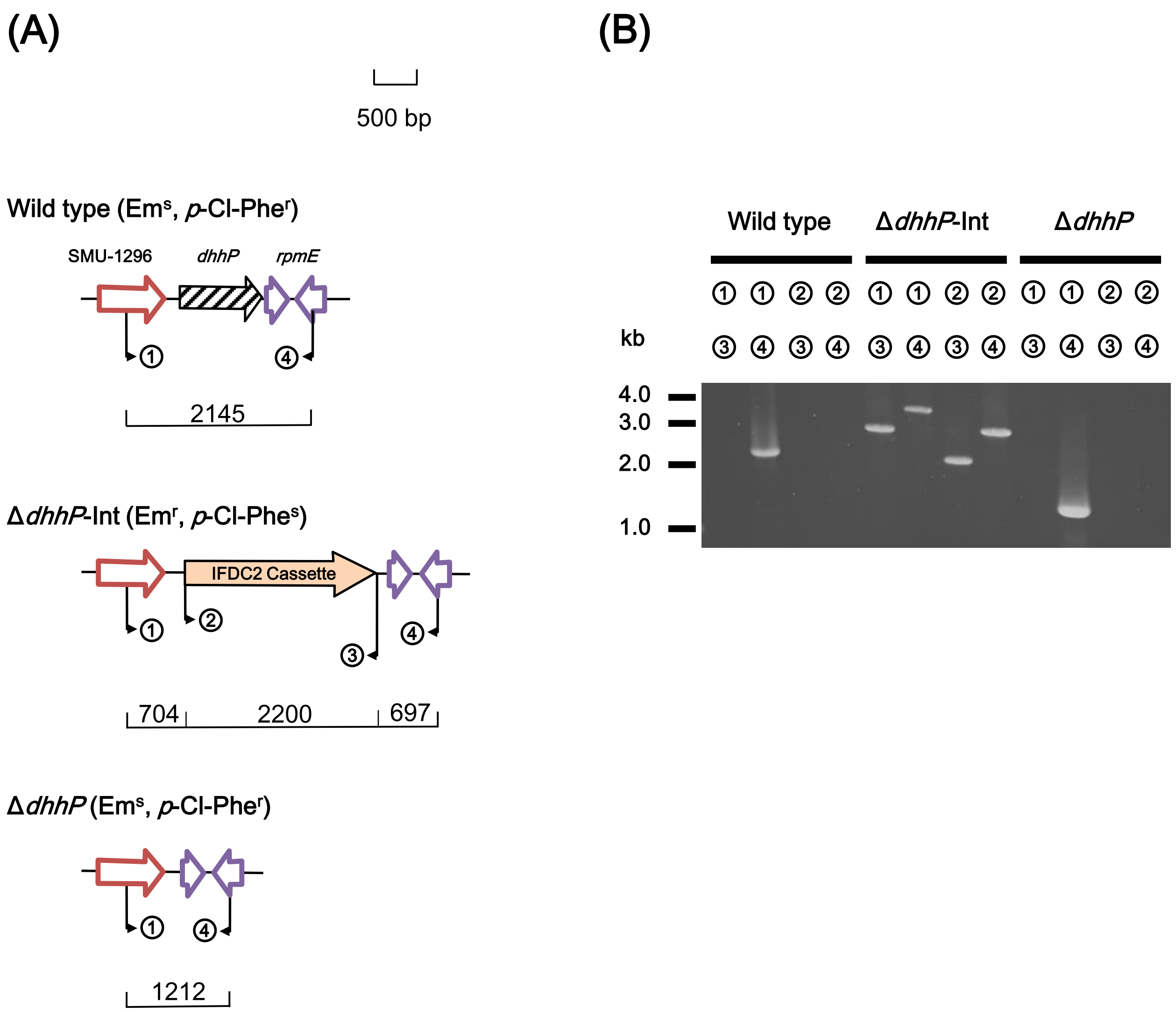

Supplemental Figure S2. Construction of a markerless mutant *S. mutans* XC lacking *dhhP*. Chromosomal gene arrangement **(A)** and verifying PCR analyses **(B)** are shown. The *dhhP* gene was initially replaced by the IFDC2 cassette of pKOdhhP-Int, yielding an intermediate mutant strain, Δ*dhhP*-Int. The resulting strain was selected using erythromycin. Subsequently, the IFDC2 cassette was replaced by a linear construct containing two linked homologous fragments without the selection cassette in pKOdhhP. The transformants were then selected on plates containing *p*-Cl-Phe. The resulting markerless mutant (Δ*dhhP*) is sensitive to erythromycin and resistant to *p*-Cl-Phe. The distances between primers are given in bp. Each circled number indicates a PCR primer used for verification. Each DNA fragment was PCR-amplified using the indicated primers. DNA size standards are shown.

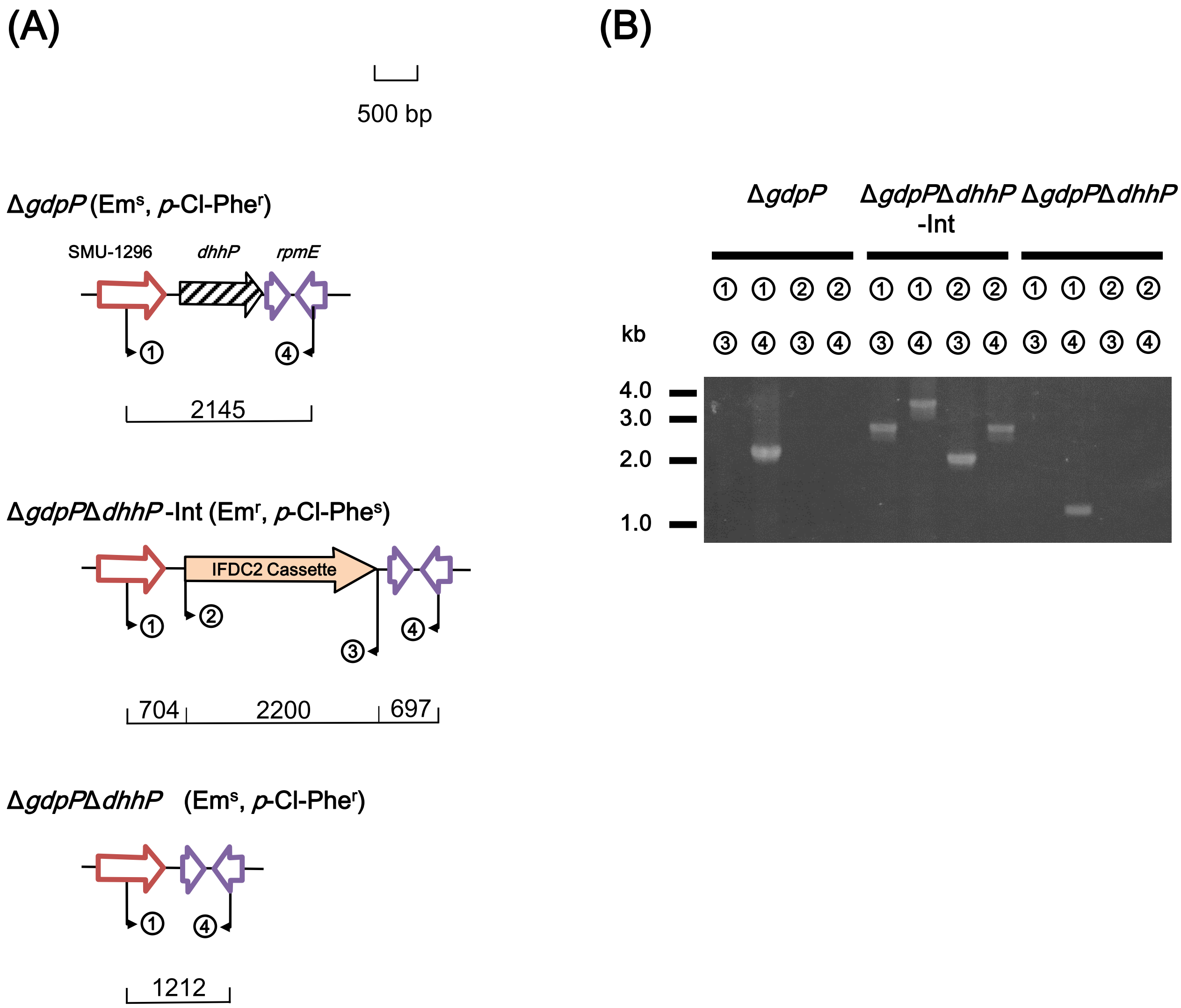

Supplemental Figure S3. Construction of a markerless double mutant *S. mutans* XC lacking both *gdpP* and *dhhP*. Chromosomal gene arrangement **(A)** and verifying PCR analyses **(B)** are shown. The *dhhP* gene in Δ*gdpP* was initially replaced by the IFDC2 cassette of pKOdhhP-Int, yielding an intermediate mutant strain, Δ*gdpP*Δ*dhhP*-Int. The resulting strain was selected using erythromycin. Subsequently, the IFDC2 cassette was replaced by a linear construct containing two linked homologous fragments without the selection cassette in pKOdhhP. The transformants were then selected on plates containing *p*-Cl-Phe. The resulting markerless mutant (Δ*gdpP*Δ*dhhP*) is sensitive to erythromycin and resistant to *p*-Cl-Phe. The distances between primers are given in bp. Each circled number indicates a PCR primer used for verification. Each DNA fragment was PCR-amplified using the indicated primers. DNA size standards are shown.
